# A Rare Case Report of Successful Laparoscopy-Guided Reduction of Non-Puerperal Uterine Inversion

**DOI:** 10.3390/medicina59040793

**Published:** 2023-04-19

**Authors:** Seongmin Kim, Sanghoon Lee, Jae-Yun Song

**Affiliations:** 1 Gynecologic Cancer Center, CHA Ilsan Medical Center, CHA University College of Medicine, 1205 Jungang-ro, Ilsandong-gu, Goyang-si 10414, Republic of Korea; naiad515@gmail.com; 2 Department of Obstetrics and Gynecology, Korea University College of Medicine, 73 Inchon-ro, Seongbuk-gu, Seoul 02841, Republic of Korea; mdleesh@gmail.com

**Keywords:** laparoscopy, uterine inversion, endometrial cancer

## Abstract

Non-puerperal uterine inversion is an extremely rare and potentially dangerous condition. Cases are poorly described in the literature, and their actual incidence is unknown. A 34-year-old nulliparous female patient visited the emergency department following a loss of consciousness. She had experienced continuous vaginal bleeding over the preceding two months, with a two-day history of worsening symptoms. The patient showed signs of hypovolemic shock secondary to unceasing vaginal bleeding. Ultrasound and computed tomography revealed an inverted uterus and a large hematoma inside the patient’s vaginal cavity. An emergency explorative laparoscopy was performed, which confirmed uterine inversion. Initially, Johnson’s maneuver was attempted under laparoscopic visualization, but this failed to achieve uterine reduction. Following the unsuccessful performance of Huntington’s maneuver, a re-trial of the manual reduction allowed the uterus to recover to its normal anatomy. The patient’s vaginal bleeding was dramatically reduced after successful uterine reduction. The pathologic report conducted confirmed endometrioid adenocarcinoma. Laparoscopic visualization is a feasible and safe procedure for achieving uterine reduction in cases of non-puerperal uterine inversion with an unconfirmed pathology. Uterine malignancies should be considered in patients with non-puerperal uterine inversion.

## 1. Introduction

Uterine inversion is a rare condition, and most cases occur during the puerperal period after a vaginal delivery [1]. However, 14% of cases are non-puerperal and, therefore, difficult to diagnose [2]. Uterine inversion can even be related to a life-threatening condition. About 170 cases have been reported, with approximately half of them attributable to submucosal fibroids. Three percent of these cases are related to endometrial carcinoma. Due to the rare incidence of a non-puerperal uterine inversion, the optimal strategy for treating and managing this condition has not yet been established. Previously reported cases showed various methods for uterine reversion. However, a successful reversion without hysterectomy is not common among such cases. In this report, we present a rare case of a successful laparoscopy-guided reversion of non-puerperal uterine inversion. A video presentation including a surgical film was also prepared as a Supplementary Material (Video S1). We expect that this research will allow clinicians to better understand the cause and clinical course of non-puerperal uterine inversion and possible treatment options from the present case report.

## 2. Case Description

A 34-year-old nulliparous woman visited our emergency department due to vaginal bleeding and chest pain. Underlying diseases included obesity (with a body mass index of 38.1) and a history of mental retardation. She was not taking any medication or undergoing any medical treatments. The parents of the patient stated that the patient barely had social contact with others. The woman had extremely late menarche, which started 6 years prior. Since menarche, she had experienced heavy menstrual bleeding every month, but she never visited a gynecological clinic to address this condition.

She had experienced continuous vaginal bleeding over the prior 2 months, and the bleeding had exacerbated 2 days before the visit. At the time of the visit, the patient showed signs of hypovolemic shock, which required intensive care with intubation and central catheter insertion. Her mental status corresponded to a state of stupor, and her vital signs showed extremely low systolic and diastolic blood pressure and tachycardia. Laboratory findings revealed extremely severe anemia (Hb 1.4 g/dL) with elevated cardiac enzymes. Transfusion was initiated immediately while preparing for a gynecological evaluation.

**Figure 1:**
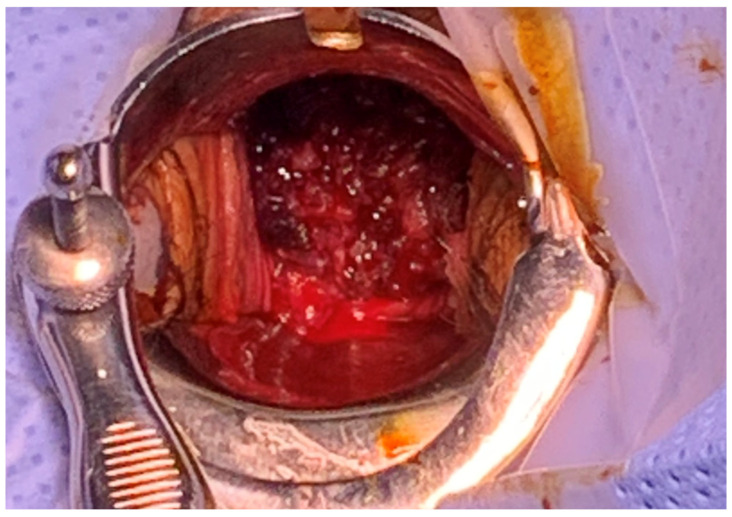
Initial vaginal examination. A solid mass-like lesion was visible along with active bleeding.

On vaginal examination, a solid mass-like lesion with an irregular surface was visible along with active bleeding (Figure 1). The uterine cervix appeared abnormal upon examination. Following transvaginal ultrasound, abnormal uterine characteristics were observed, but the bilateral ovaries were well-defined. Following computed tomography, a definite sign of uterine inversion was suspected, without demonstrable vaginal tumors. Immediately following the diagnosis of non-puerperal uterine inversion, Johnson’s maneuver was attempted to perform uterine reversion at bedside. However, despite several attempts at manual replacement, no relaxation or mobility was obtained when traction was applied. The extent of vaginal bleeding had not been reduced at all, and hypotension was not recovered quickly even after extensive transfusion and the provision of massive fluid supply. Further treatment with surgical management was warranted to explore the severity of the inversion and determine a method of reducing the patient’s vaginal bleeding.

**Figure 2:**
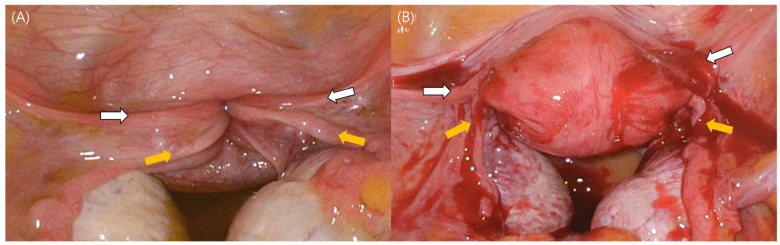
Laparoscopic visualization of non-puerperal uterine inversion. The classic “flowerpot” appearance can be seen. White arrows indicate both round ligaments, while yellow arrows show bilateral fallopian tubes. (**A**) During laparoscopy, a second-degree uterine inversion was confirmed. (**B**) Laparoscopic visualization of normal-sized uterus with smooth surface.

An emergency laparoscopic exploration was prepared and performed, and second-degree uterine inversion was confirmed (Figure 2A). The classic “flowerpot” appearance was observed, namely, a complete inversion with the involvement of bilateral round ligaments and fallopian tubes. Initially, the sponge-on-a-stick method was used to attempt Johnson’s maneuver, whereby the uterine fundus is pushed through the cervical ring toward the umbilicus. However, uterine reversion was not achieved. Huntington’s maneuver was then attempted, during which the round ligaments were identified, and upward traction was applied to them, while a sponge on a stick inserted in the vagina was used to push the inverted uterus upwards; however, this was not successful either. A re-trial of Johnson’s maneuver with manual reduction using the surgeons’ fingers allowed the uterus to recover its normal physical characteristics (Figure 2B). The patient’s vaginal bleeding dramatically reduced, and a normal uterine cervix was identified after reversion. After reversion to normal uterine anatomy, manual intrauterine examination was performed, revealing a 2 cm sized, round, hard mass lesion. The mass was manually removed from the uterus and sent to the pathological department. The pathological report confirmed an endometrioid adenocarcinoma of the endometrium.

The postoperative course of the patient was stable, showing only a small amount of uterine bleeding. Magnetic resonance imaging (MRI) was performed 1 week following surgery. The MRI identified endometrial carcinoma without myometrial invasion or lymphadenopathy.

After a few weeks of patient recovery and disease evaluation, a robotically assisted laparoscopic staging operation including a total hysterectomy, a bilateral salpingo-oophorectomy, and bilateral pelvic sentinel lymph node mapping was conducted. On the final pathological report, a stage-Ia low-grade endometrioid adenocarcinoma of the endometrium was confirmed. There was no sentinel lymph node invasion. On follow-up, the patient demonstrated no evidence of disease, and this remains the case to date.

## 3. Discussion

To the best of our knowledge, this is the first study including videos supplements reporting non-puerperal uterine inversion. In addition, no other reports exist regarding a successful laparoscopy-assisted reduction in inversion induced by a uterine carcinoma. Six cases had been previously reported to present uterine inversion associated with endometrial carcinoma (Table 1). All the cases were managed by laparotomy, including subtotal hysterectomy, total abdominal hysterectomy, and radical hysterectomy [3,4,5,6,7,8]. Surprisingly, uterine reversion was only successful in one laparotomy case. Additionally, the trial of uterine reversion failed in two cases during surgery. The present case is distinguished by successful uterine reversion achieved via a laparoscopic approach. A traditional approach in which laparotomy is used to manage non-puerperal inversion arising from an endometrial carcinoma would require a longer period of recovery after surgery. On the other hand, a laparoscopic approach has the benefit of reduced postoperative pain and faster recovery. We believe that this report will contribute to the literature by showing the feasibility of the laparoscopically assisted management of non-puerperal inversion.

On the other hand, there were five previous cases of non-puerperal uterine inversion that were managed by a laparoscopic approach (Table 2) [9,10,11,12,13]. The expulsion of submucosal leiomyoma was found to be the cause in all cases. Among them, successful uterine reduction was achieved in two via the Haultain procedure, wherein the cervical ring was opened anteriorly or posteriorly. Reversion was achieved, followed by the repair of uterine incision. In this case, endometrial cancer was diagnosed from a biopsy, and a uterine reversion was conducted via Johnson’s maneuver.

The suspected mechanism of non-puerperal inversion is as follows. If a tumor in the uterine fundus grows, uterine contraction can occur, thereby expelling the tumor outside the uterus. When the tumor is expelled from the cervical os, the uterus and tumor pass through the vagina.

In this case report, the patient eventually underwent total hysterectomy to treat her uterine malignancy. The patient was originally advised to try a fertility-sparing treatment including a comprehensive progestin regimen. However, the patient had a condition of mental retardation, rendering compliance unexpected for a long-duration treatment. Her guardians, that is, her parents, requested that we perform surgical treatment to arrive at a better prognosis regarding the patient’s condition. Clinicians should consider and engage in consultation about fertility-sparing options for the treatment of early-stage endometrial carcinoma.

In conclusion, laparoscopic visualization is a feasible and safe procedure for the reversion of non-puerperal uterine inversion. Optimal treatment strategies must be further elucidated due to the rarity of this disease. A uterine malignancy should be considered when assessing patients with non-puerperal uterine inversion.

## Figures and Tables

**Table 1:** Previously reported cases of uterine inversion associated with endometrial carcinoma.

Case	Age	Image	Approach	Reduction	Management
Kumar (2005) [3]	51	MRI	Laparotomy	No	Radical hysterectomy, BSO, PLND
Moulding (2004) [4]	52	MRI	Laparotomy	No	Total hysterectomy
Oguri (2005) [5]	60	MRI	Laparotomy	Yes	Total hysterectomy, BSO, PLND
Prefontaine M (2012) [6]	71	CT	Laparotomy	No	Total hysterectomy, BSO
Simms-stewart (2008) [7]	78	US (failed)	Laparotomy	Failed	Subtotal hysterectomy
Ueda (2006) [8]	28	CT	Laparotomy	Failed	Radical hysterectomy, BSO, PLND
Current case	34	CT	Laparoscopy	Yes	Reduction and delayed surgery (robotically assisted total laparoscopic hysterectomy, BSO, sentinel lymph node mapping)

BSO, bilateral salpingo-oophorectomy; CT, computed tomography; MRI, magnetic resonance imaging; PLND, pelvic lymph node dissection; US, ultrasound.

**Table 2:** Uterine inversion managed by laparoscopic approach.

Case	Age	Image	Diagnosis	Reduction	Management
Auber (2011) [9]	40	US	Leiomyoma	No	Total hysterectomy
Minas (2015) [10]	47	MRI	Leiomyoma	No	Total hysterectomy
Zhang (2015) [11]	34	MRI	Leiomyoma	Yes (Haultain procedure)	Myomectomy
Arpita (2019) [12]	38	MRI	Leiomyoma	Yes (Haultain procedure)	Total hysterectomy
Kinza (2019) [13]	47	US	Leiomyoma	No	Total hysterectomy
Current case	34	CT	Endometrial carcinoma	Yes (Johnson’s maneuver)	Reduction and delayed surgery (robotically assisted total laparoscopic hysterectomy, BSO, sentinel lymph node mapping)

BSO, bilateral salpingo-oophorectomy; CT, computed tomography; MRI, magnetic resonance imaging; US, ultrasound.

## Data Availability

The data presented in this study are available on request from the corresponding author.

## Supplementary Materials

The following supporting information can be downloaded at: https://www.mdpi.com/article/10.3390/medicina59040793/s1, Video S1: Video presentation of a rare case report of successful laparoscopy-guided reduction of non-puerperal uterine inversion.
